# Draft Genome Sequence of *Serratia* sp. Strain ATCC 39006, a Model Bacterium for Analysis of the Biosynthesis and Regulation of Prodigiosin, a Carbapenem, and Gas Vesicles

**DOI:** 10.1128/genomeA.01039-13

**Published:** 2013-12-12

**Authors:** Peter C. Fineran, Marina C. Iglesias Cans, Joshua P. Ramsay, Nabil M. Wilf, Desiree Cossyleon, Matthew B. McNeil, Neil R. Williamson, Rita E. Monson, S. Anette Becher, Jo-Ann L. Stanton, Kim Brügger, Steven D. Brown, George P. C. Salmond

**Affiliations:** Department of Microbiology and Immunology, University of Otago, Dunedin, New Zealanda; Department of Biochemistry, University of Cambridge, Cambridge, United Kingdomb; AgResearch Ltd., Invermay Agricultural Centre, Mosgiel, New Zealandc; Department of Anatomy, University of Otago, Dunedin, New Zealandd; EASIH, University of Cambridge, Addenbrooke’s Hospital, Cambridge, United Kingdome; Biosciences Division, Oak Ridge National Laboratory, Oak Ridge, Tennessee, USAf

## Abstract

*Serratia* sp. strain ATCC 39006 is a Gram-negative bacterium and a member of the *Enterobacteriaceae* that produces various bioactive secondary metabolites, including the tripyrrole red pigment prodigiosin and the β-lactam antibiotic 1-carbapenen-2-em-3-carboxylic acid (a carbapenem). This strain is the only member of the *Enterobacteriaceae* known to naturally produce gas vesicles, as flotation organelles. Here we present the genome sequence of this strain, which has served as a model for analysis of the biosynthesis and regulation of antibiotic production.

## GENOME ANNOUNCEMENT

*Serratia* sp. strain ATCC 39006 was originally isolated from *Salicornia alterniflora* and in channel water from a salt marsh in Cheesequake, NJ, in a search by the Squibb Chemical Company for bacteria producing new antibiotics (1). In addition to the β-lactam produced, identified as 1-carbapen-2-em-3-carboxylic acid (a carbapenem) (2), this strain synthesizes the red, linear tripyrrole pigment prodigiosin (2-methyl-3-pentyl-6-methoxyprodigiosin). Prodigiosin is a secondary metabolite with antimicrobial, anticancer, and immunosuppressant properties with derivatives in clinical trials (3, 4). *Serratia* sp. strain ATCC 39006 was used to determine the prodigiosin biosynthetic pathway, with implications for biosynthesis of the related compound, undecylprodigiosin, produced by *Streptomyces coelicolor* (4, 5). Furthermore, *Serratia* sp. strain ATCC 39006 has provided an excellent model for investigating the regulation of antibiotic biosynthesis in Gram-negative enterobacteria (4). The control of these secondary metabolites is complex and responds to quorum sensing (6–8), cyclic di-GMP signaling (9, 10), phosphate availability (7, 11), carbon source (12), Hfq (13), stationary phase (14), and drug efflux pump activity (15), among other factors. In addition, due to the ease of prodigiosin detection, this strain has been used to analyze conserved uncharacterized genes and gene products (16–18). For example, SdhE was recently investigated in this strain. SdhE is widely conserved in eukaryotes and *Alpha*-, *Beta*-, and *Gammaproteobacteria* and is essential for flavinylation and activation of succinate dehydrogenase, an enzyme central to the electron transport chain and the tricarboxylic acid cycle (17, 19, 20).

*Serratia* sp. strain ATCC 39006 is motile by means of flagella and can swarm over surfaces aided by the production of a biosurfactant (10). Surprisingly, this strain also produces gas vesicles, which are hollow intracellular proteinaceous organelles that control bacterial buoyancy and allow flotation toward air-liquid interfaces (21). This is the only known enterobacterium to utilize this form of taxis naturally (21). The secretion of plant cell wall-degrading enzymes is also a feature of this bacterium, and plant pathogenicity has been confirmed in potato tuber-rotting assays (6, 9). Furthermore, this strain is virulent in a *Caenorhabditis elegans* infection model (22). The genetic analysis of *Serratia* sp. strain ATCC 39006 has been greatly facilitated by the isolation of an efficient broad-host-range generalized transducing phage (23).

Genomic DNA of *Serratia* sp. strain ATCC 39006 was sequenced using the 454 GS FLX Titanium platform (Roche) (~18× coverage single-end data) and 36-bp Illumina single-end reads (GAIIx) (~439× coverage). The 454 data were *de novo* assembled (Newbler v2.3), giving 53 large contigs (99.9% of sequence) from 94 total contigs. These were assembled into 5 scaffolds using PCR and Sanger sequencing (3 contigs between 200 and 1,000 bp remained). Illumina reads were mapped using BWA 0.5.8, indels were detected using GATK (24), and the sequence was polished using a custom perl script.

The *Serratia* sp. strain ATCC 39006 genome is ~4.94 Mb (G+C content of 49.2%), with 4,413 protein-encoding genes, 7 rRNA operons, and 72 tRNAs (predicted using Prodigal [25]). This sequence will now enable further analysis of the diverse and interesting biological traits that have been defined in this unusual enterobacterium.

### Nucleotide sequence accession numbers.

This whole-genome shotgun project has been deposited at DDBJ/EMBL/GenBank under the accession number AWXH00000000. The version described in this paper is version AWXH01000000.

## References

[1] Parker WL Rathnum ML Wells JS Jr Trejo WH Principe PA Sykes RB 1982 SQ 27,860, a simple carbapenem produced by species of *Serratia* and *Erwinia*. J. Antibiot. 35:653–660 7118721 10.7164/antibiotics.35.653

[2] Coulthurst SJ Barnard AM Salmond GP 2005 Regulation and biosynthesis of carbapenem antibiotics in bacteria. Nat. Rev. Microbiol. 3:295–306 15759042 10.1038/nrmicro1128

[3] Williamson NR Fineran PC Gristwood T Chawrai SR Leeper FJ Salmond GP 2007 Anticancer and immunosuppressive properties of bacterial prodiginines. Future Microbiol. 2:605–618 18041902 10.2217/17460913.2.6.605

[4] Williamson NR Fineran PC Leeper FJ Salmond GP 2006 The biosynthesis and regulation of bacterial prodiginines. Nat. Rev. Microbiol. 4:887–899 17109029 10.1038/nrmicro1531

[5] Williamson NR Simonsen HT Ahmed RA Goldet G Slater H Woodley L Leeper FJ Salmond GP 2005 Biosynthesis of the red antibiotic, prodigiosin, in *Serratia*: identification of a novel 2-methyl-3-n-amyl-pyrrole (MAP) assembly pathway, definition of the terminal condensing enzyme, and implications for undecylprodigiosin biosynthesis in *Streptomyces*. Mol. Microbiol. 56:971–989 15853884 10.1111/j.1365-2958.2005.04602.x

[6] Fineran PC Slater H Everson L Hughes K Salmond GP 2005 Biosynthesis of tripyrrole and beta-lactam secondary metabolites in *Serratia*: integration of quorum sensing with multiple new regulatory components in the control of prodigiosin and carbapenem antibiotic production. Mol. Microbiol. 56:1495–1517 15916601 10.1111/j.1365-2958.2005.04660.x

[7] Slater H Crow M Everson L Salmond GP 2003 Phosphate availability regulates biosynthesis of two antibiotics, prodigiosin and carbapenem, in *Serratia* via both quorum-sensing-dependent and -independent pathways. Mol. Microbiol. 47:303–320 12519208 10.1046/j.1365-2958.2003.03295.x

[8] Thomson NR Crow MA McGowan SJ Cox A Salmond GP 2000 Biosynthesis of carbapenem antibiotic and prodigiosin pigment in *Serratia* is under quorum sensing control. Mol. Microbiol. 36:539–556 10844645 10.1046/j.1365-2958.2000.01872.x

[9] Fineran PC Williamson NR Lilley KS Salmond GP 2007 Virulence and prodigiosin antibiotic biosynthesis in *Serratia* are regulated pleiotropically by the GGDEF/EAL domain protein, PigX. J. Bacteriol. 189:7653–7662 17766413 10.1128/JB.00671-07 PMC2168757

[10] Williamson NR Fineran PC Ogawa W Woodley LR Salmond GP 2008 Integrated regulation involving quorum sensing, a two-component system, a GGDEF/EAL domain protein and a post-transcriptional regulator controls swarming and RhlA-dependent surfactant biosynthesis in *Serratia*. Environ. Microbiol. 10:1202–1217 18294208 10.1111/j.1462-2920.2007.01536.x

[11] Gristwood T Fineran PC Everson L Williamson NR Salmond GP 2009 The PhoBR two-component system regulates antibiotic biosynthesis in *Serratia* in response to phosphate. BMC Microbiol. 9:112 19476633 10.1186/1471-2180-9-112 PMC2695467

[12] Fineran PC Everson L Slater H Salmond GP 2005 A GntR family transcriptional regulator (PigT) controls gluconate-mediated repression and defines a new, independent pathway for regulation of the tripyrrole antibiotic, prodigiosin, in *Serratia*. Microbiology 151:3833–3845 16339930 10.1099/mic.0.28251-0

[13] Wilf NM Williamson NR Ramsay JP Poulter S Bandyra KJ Salmond GP 2011 The RNA chaperone, Hfq, controls two luxR-type regulators and plays a key role in pathogenesis and production of antibiotics in *Serratia* sp. ATCC 39006. Environ. Microbiol. 13:2649–2666 21824244 10.1111/j.1462-2920.2011.02532.x

[14] Wilf NM Salmond GP 2012 The stationary phase sigma factor, RpoS, regulates the production of a carbapenem antibiotic, a bioactive prodigiosin and virulence in the enterobacterial pathogen *Serratia* sp. ATCC 39006. Microbiology 158:648–658 22194349 10.1099/mic.0.055780-0

[15] Gristwood T Fineran PC Everson L Salmond GP 2008 PigZ, a TetR/AcrR family repressor, modulates secondary metabolism via the expression of a putative four-component resistance-nodulation-cell-division efflux pump, ZrpADBC, in *Serratia* sp. ATCC 39006. Mol. Microbiol. 69:418–435 18485072 10.1111/j.1365-2958.2008.06291.x

[16] Gristwood T McNeil MB Clulow JS Salmond GP Fineran PC 2011 PigS and PigP regulate prodigiosin biosynthesis in *Serratia* via differential control of divergent operons, which include predicted transporters of sulfur-containing molecules. J. Bacteriol. 193:1076–1085 21183667 10.1128/JB.00352-10 PMC3067589

[17] McNeil MB Clulow JS Wilf NM Salmond GP Fineran PC 2012 SdhE is a conserved protein required for flavinylation of succinate dehydrogenase in bacteria. J. Biol. Chem. 287:18418–18428 22474332 10.1074/jbc.M111.293803 PMC3365757

[18] McNeil MB Iglesias-Cans MC Clulow JS Fineran PC 2013 YgfX (CptA) is a multimeric membrane protein that interacts with the succinate dehydrogenase assembly factor SdhE (YgfY). Microbiology 159:1352–1365 23657679 10.1099/mic.0.068510-0

[19] McNeil MB Fineran PC 2013 Prokaryotic assembly factors for the attachment of flavin to complex II. Biochim. Biophys. Acta 1827:637–647 22985599 10.1016/j.bbabio.2012.09.003

[20] McNeil MB Fineran PC 2013 The conserved RGxxE motif of the bacterial FAD Assembly factor SdhE is required for succinate dehydrogenase flavinylation and activity. Biochemistry 52:7628–7640 24070374 10.1021/bi401006a

[21] Ramsay JP Williamson NR Spring DR Salmond GP 2011 A quorum-sensing molecule acts as a morphogen controlling gas vesicle organelle biogenesis and adaptive flotation in an enterobacterium. Proc. Natl. Acad. Sci. U. S. A. 108:14932–14937 21873216 10.1073/pnas.1109169108 PMC3169117

[22] Coulthurst SJ Kurz CL Salmond GP 2004 *luxS* mutants of *Serratia* defective in autoinducer-2-dependent “quorum sensing” show strain-dependent impacts on virulence and production of carbapenem and prodigiosin. Microbiology 150:1901–1910 15184576 10.1099/mic.0.26946-0

[23] Evans TJ Crow MA Williamson NR Orme W Thomson NR Komitopoulou E Salmond GP 2010 Characterization of a broad-host-range flagellum-dependent phage that mediates high-efficiency generalized transduction in, and between, *Serratia* and *Pantoea*. Microbiology 156:240–247 19778959 10.1099/mic.0.032797-0

[24] McKenna A Hanna M Banks E Sivachenko A Cibulskis K Kernytsky A Garimella K Altshuler D Gabriel S Daly M DePristo MA 2010 The Genome Analysis Toolkit: a MapReduce framework for analyzing next-generation DNA sequencing data. Genome Res. 20:1297–1303 20644199 10.1101/gr.107524.110 PMC2928508

[25] Hyatt D Chen GL Locascio PF Land ML Larimer FW Hauser LJ 2010 Prodigal: prokaryotic gene recognition and translation initiation site identification. BMC Bioinformatics 11:119 20211023 10.1186/1471-2105-11-119 PMC2848648

